# Evaluating the Factor Structure of the Emotion Dysregulation Scale-Short (EDS-s): A Preliminary Study

**DOI:** 10.3390/ijerph19010418

**Published:** 2021-12-31

**Authors:** Giulia Raimondi, Claudio Imperatori, Mariantonietta Fabbricatore, David Lester, Michela Balsamo, Marco Innamorati

**Affiliations:** 1 Department of Human Sciences, European University of Rome, 00163 Rome, Italy; claudio.imperatori@unier.it (C.I.); Mariantonietta.fabbricatore@unier.it (M.F.); marco.innamorati@unier.it (M.I.); 2 Psychology Program, Stockton University, Galloway, NJ 08205, USA; david.lester@stockton.edu; 3 Psychological, Health, and Territorial Sciences, G. d’Annunzio University Chieti–Pescara, 66100 Chieti, Italy; michela.balsamo@unich.it

**Keywords:** emotion dysregulation, structural equation modelling, binge eating, general psychopathology

## Abstract

Emotion dysregulation (ED) can be considered a psychopathological transdiagnostic dimension, the presence of which should be reliably screened in clinical settings. The aim of the current study was to validate the Italian version of the Emotion Dysregulation Scale-short (EDS-s), a brief self-report tool assessing emotion dysregulation, in a non-clinical sample of 1087 adults (768 women and 319 men). We also assessed its convergent validity with scales measuring binge eating and general psychopathology. Structural equation modeling suggested the fit of a one-factor model refined with correlations between the errors of three pairs of items (χ^2^ = 255.56, df = 51, *p* < 0.001, RMSEA = 0.08, CFI = 0.94, TLI = 0.93, SRMR = 0.04). The EDS-s demonstrated satisfactory internal consistency (ordinal alpha = 0.94). Moreover, EDS-s scores partly explained the variance of both binge eating (0.35, *p* < 0.001) and general psychopathology (0.60, *p* < 0.001). In conclusion, the EDS-s can be considered to be a reliable and valid measure of ED.

## 1. Introduction

Emotion regulation can be defined as the ability to correctly identify, monitor, express, and modulate the intensity of emotions [1], and emotion dysregulation (ED) is a difficulty or inability to carry out this process. ED could be associated with deficits in: (i) the comprehension, awareness, and acceptance of the emotion; (ii) the ability to modulate one’s emotional response; and (iii) the ability to use emotions in one’s goal-directed behaviors [2]. There is an overall consensus about considering ED as a transdiagnostic feature characterizing several disorders [3]. For example, deficits in emotion regulation have been reported in several conditions, including eating disorders [4], mood and anxiety disorders [5], sleep disorders [6], psychotic disorders [7], personality disorders [8], and dissociative disorders [9].

Several self-report instruments assessing ED have been proposed in recent years, including the Difficulties in Emotion Regulation Scale (DERS) [2] and the Emotion Regulation Questionnaire (ERQ) [10]. The DERS is a 36-item multidimensional self-report questionnaire measuring six dimensions of difficulties in emotion regulation: (1) Non-acceptance, referring to the individual’s non-acceptance of his/her emotions; (2) Goals, related to the difficulty in carrying out goal-directed behaviors while experiencing negative emotions; (3) Impulse, referring to the individual’s difficulty in controlling his/her impulses when experiencing negative emotions; (4) Awareness, related to the individual’s awareness of the emotions he/she is experiencing; (5) Strategies, related to the emotion-regulation strategies chosen by the individual to modulate his/her emotional response; and (6) Clarity, related to the lack of emotional clarity. Although Gratz and Roemer [2] reported satisfactory psychometric properties (e.g., Cronbach’s α of 0.93 for total score, Cronbach’s α > 0.80 for each subscale, and good predictive validity with behaviors associated with ED, such as self-harm and marital violence), research has reported contradictory results (e.g., favoring a five-factor model rather than the original six-factor model) [11,12,13].

The Emotion Regulation Questionnaire (ERQ) [10] measures two emotion regulation strategies, namely, Cognitive Reappraisal, which refers to the change in interpretation of an emotionally-triggering situation with the aim of changing its emotional impact, and Expressive Suppression, which refers to the inhibition of the emotional response. It is composed of 10 items, rated on a 7-point Likert scale (from 1 = strongly disagree to 7 = strongly agree). Although adequate psychometric properties have been observed in different studies in university student samples (e.g., a good fit for the hypothesized two-factor model and Cronbach’s α > 0.70 for both subscales) [14,15], Spaapen et al. [16] and Wiltink et al. [17] were unable to confirm the original factor structure in community samples. This highlights the need for validating the ERQ in a sample representative of the general population rather than in university students [16]. Moreover, Spaapen et al. [16] suggested excluding item #3.

Considering the limitations of existing measures of ED, Powers et al. [18] proposed the Emotion Dysregulation Scale (EDS-s), a 12-item questionnaire derived from a previous unpublished and longer questionnaire (see [19]). In the validation study, the EDS scores were significantly associated with all the subscales of the DERS, and the EDS demonstrated incremental validity over the DERS in predicting different psychopathology conditions (e.g., substance abuse symptoms, post-traumatic stress symptoms, borderline pathology, depressive symptoms, and number of suicide attempts) [18]. Other studies have supported the use of the EDS in a variety of clinical populations [20,21,22,23].

Although the EDS-s could be a promising tool for assessing ED, its psychometric properties have not been thoroughly investigated. For example, no studies have carried out a confirmative factorial analysis (CFA) to investigate and support the unidimensionality of the EDS-s. Although the items in the EDS-s were selected from items with the highest factor loadings from a longer version of the questionnaire, this procedure is not sufficient to guarantee the unidimensionality of the scale [24] and does not exclude the possible presence of other issues associated with the dimensionality of the scale (e.g., the presence of locally dependent items). Therefore, the aim of the current study was to investigate the factor structure of an Italian version of the EDS-s in a nonclinical sample of adults recruited from the general population. By means of structural equation modeling (SEM), we tested the adequacy of the one-factor structure and the convergent validity of the EDS-s in relation to measures of binge eating severity and general psychopathology.

## 2. Materials and Methods

### 2.1. Participants

A convenience sample of 1087 Italian adults (768 (70.7%) women and 319 (29.3%) men; mean age: 36.02, SD = 15.56 years) participated in the study. The inclusion criteria were an age of 18 years and older and the ability to complete the assessment. The exclusion criterion was the inability to complete the assessment for any reason, including refusal of informed consent. The sample was recruited through advertisements at universities campuses and on social media (e.g., flyers and online ads). The questionnaires were completed using a Google form, and personal identifiable information was not collected. Participants were administered the study protocol between May 2017 and November 2020. Sociodemographic and clinical characteristics of the sample are reported in Table 1.

All participants agreed to take part in the study voluntarily and provided written informed consent. Participants did not receive any payment or other compensation (e.g., academic credits for university students). The study was approved by the Ethics Committee of the European University of Rome (Rome, Italy) (on the 26 June 2021), and was performed according to the Helsinki declaration standards.

### 2.2. Measures

All the participants were administered a checklist assessing socio-demographic variables (i.e., age, sex, marital status, education level, occupation) and clinical variables (i.e., height, weight, use of tobacco, alcohol, and “legal highs”), and the Italian version of the EDS-s [18]. Finally, 295 participants were also administered the Italian versions of the Binge Eating Scale (BES) [25] and the Brief Symptoms Inventory (BSI) [26]. The measures analyzed in this study were part of a larger protocol and, in order to reduce the burden of the assessment, only the EDS-s was administered to the whole sample. The BES and the BSI were administered to a subsample of 295 individuals. According to Wolf et al. [27], a minimum of 200 participants is required for regression models with SEM. Moreover, Bentler and Chou [28] suggested a number of 10 observations for each estimated parameter. Therefore, our study has a satisfactory power to detect a significant effect. Sociodemographic and clinical characteristics of the sample are reported in Table 2.

### 2.3. Emotion Dysregulation

The EDS-s [18] is a 12-item self-report measure which assesses emotional (“Emotions overwhelm me”), cognitive (“When I am feeling bad, I have trouble remembering anything positive, everything just seems bad”), and behavioral (“When my emotions are strong, I often make bad decisions”) features of emotion dysregulation. Respondents are asked to rate each item on a 7-point Likert scale (from 1 = “Not true”, to 7 = “Very true”), with higher scores reflecting higher emotional dysregulation. The Italian version of the EDS-s, used in this study, was obtained using the back-translation procedure. One of the authors (GR) translated the scale into Italian, and a second author (CI) performed the back-translation. To ensure the accurate translation of the scale, a third author (MI) checked for the presence of errors and ambiguities (see Appendix A for the Italian version of the EDS-s).

### 2.4. Binge Eating Severity

The BES [29] is a 16-item self-report questionnaire assessing binge eating severity through both behavioral and cognitive/emotional features of binge eating. For each item, participants are required to choose between three or four response statements of increasing severity. The total score ranges between 0 and 46, with a cut-off score of <18 discriminating individuals with and without binge eating symptoms. Cronbach’s α in the present sample was 0.87.

### 2.5. General Psychopathology

The BSI [30] is a self-report questionnaire assessing general psychopathology derived from the longer Symptom Checklist 90-Revised (SCL-90-R) [31]. The BSI is composed of 53 items rated on a 5-point Likert scale (from 0 = “Not at all” to 4 = “Extremely”), assessing the level of distress for the past seven days on nine psychopathological symptomatic dimensions (i.e., somatization, obsession–compulsion, interpersonal sensitivity, depression, anxiety, hostility, phobic anxiety, paranoid ideation, and psychoticism). The Global Severity Index (GSI), reflecting the severity of general psychopathology, is given by the sum of all the items. Cronbach’s α in the present sample was 0.95.

### 2.6. Statistical Analysis

All the analyses were performed with Mplus 8.3 (Los Angeles, CA: Muthén & Muthén) [32], R [33], and the Statistical Package for the Social Sciences (SPSS 25, Armonk, NY: IBM Corp) [34].

In order to analyze the factor structure of the EDS, the sample was split randomly into two subsamples (first subsample, *n* = 541; second subsample, *n* = 546). Bartlett’s test of sphericity and the Kaiser–Meyer–Olkin (KMO) test were performed on both the subsamples to assess whether the data were suitable for factor analysis. Adequacy of the correlation matrix is suggested by a significant Bartlett’s test (*p* < 0.05) and a KMO index > 0.70. A CFA with a single latent factor, using a mean and variance-adjusted weighted least square (WLSMV) estimator with a polychoric correlation matrix was performed on the first subsample, and the fit of the model was evaluated using the following indices: (1) the root mean square error of approximation (RMSEA), with values below 0.05 indicating evidence of absolute fit, values between 0.05 and 0.08 indicating the adequacy of the model, and values above or equal to 0.10 indicating the poor fit of the model [35,36]; (2) the Tucker–Lewis Index (TLI), with values > 0.95 indicating the good fit of the model and values of 0.90 and higher an acceptable fit; (3) the Comparative Fit Index (CFI), with values > 0.95 indicating good model fit and values of 0.90 and higher an acceptable fit; (4) the standardized root mean square residual (SRMR), with values <0.08 indicating good fit [37]; and (5) the chi-square (χ^2^) test, with *p*-values greater than 0.05 indicating an adequate fit to the data. However, χ^2^ is sensitive to sample size, and so *p*-values might become significant for large samples [38]. In case of suboptimal fit of the model, large modification indices (>10) were inspected to suggest refinements to add to the model. Modification indices (MIs) may suggest the need to add a path between variables or constraint/free one or more parameters. Values greater than 10 indicate that the model would be improved if a modification index is applied, and the *p*-value for the new parameter would be <0.001. However, since refinement of the factor model based on MIs is a data driven approach, modification indices should be considered one by one, and the model should be tested each time. To support the dimensionality of the EDS-s, an exploratory factor analysis (EFA) with a weighted least square estimator (WLS) was performed on the second subsample, and eigenvalues were calculated. The Kaiser–Guttman rule (factors with eigenvalues >1) [39] and eventually a parallel analysis was used to decide on the dimensionality of the scale. A final CFA, based on the results from the modification indices and supported by the results of the EFA, was performed on the first subsample, and the model fit was evaluated with the same indices reported above.

Indices of internal consistency (i.e., ordinal alpha, the Molenaar Sijtsma statistic (MS), and latent class reliability coefficient (LCRC)) were calculated, and tests evaluating differences among sex- and age-groups were performed on the whole sample (*n* = 1087). A *t*-test was used to analyze sex differences. To assess age differences, we categorized participants in three age groups, namely, young adults (18–34), adults (35–49) and late adults (50–69), and we performed an analysis of variance (ANOVA) with post hoc Bonferroni correction.

Finally, the convergent validity of the EDS-s with binge eating severity and general psychopathology was evaluated by means of a regression model using the structural equation model (SEM) approach. Bootstrap confidence intervals of 99% (99% CI; with 5000 bootstrap samples) [40] were calculated. The sub-sample used for the regression analysis was composed of the 295 participants who completed the protocol.

## 3. Results

### 3.1. Dimensionality of the EDS

The correlation matrices of both subsamples were adequate for factor analysis (first subsample: Bartlett’s test of sphericity = 3957.62; df = 66; *p* < 0.001; KMO = 0.93; second subsample: Bartlett’s test of sphericity = 3777.66; df = 66; *p* < 0.001; KMO = 0.93). The model with a single latent factor did not fit the data (RMSEA = 0.13; CFI = 0.87; TLI = 0.84; SRMR = 0.05). However, an EFA indicated the presence of only one factor with an eigenvalue >1 (=6.57), explaining 54% of the variance of the data.

**Figure 1:**
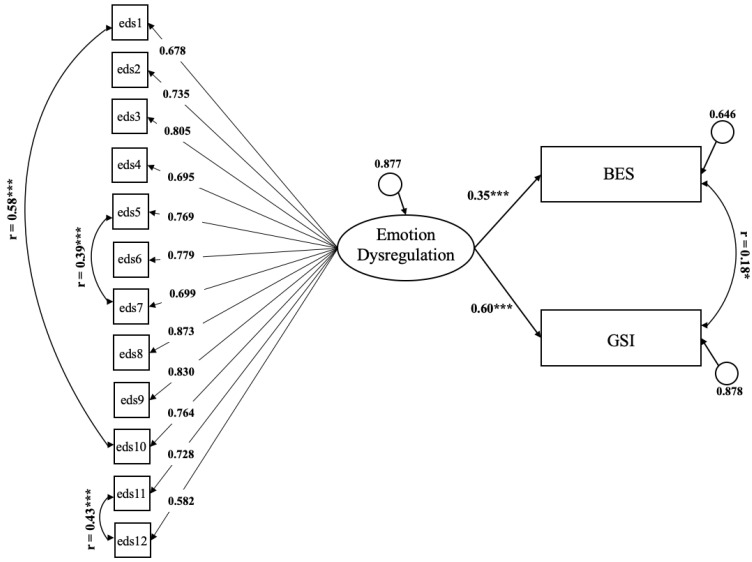
SEM Regression Analysis of the refined EDS-s with binge eating symptomatology (BES) and general psychopathology (GSI) as the dependent variables. * *p* < 0.05; *** *p* < 0.001.

MI suggested some refinements to add to the one-factor model. Specifically, the inclusion of paths between three pairs of items’ errors (item #1 and #10, item #11 and #12, and item #5 and #7) was associated with the highest estimated parameter change (0.75, 0.53, and 0.66, respectively), and significant (*p* < 0.001) changes in the value of the χ^2^ statistic. The decision to add these paths was also supported theoretically, since the pairs of items have similar meanings (item #1, “It’s often hard for me to calm down when I’m upset” and #10, “I have trouble soothing myself when I am upset”; item #12, “When my emotions are strong, I often make bad decisions” and #11, “When my emotions are stirred up, I have trouble thinking clearly”; and item #7, “When I’m upset, I have trouble remembering that people care about me” and #5, “When I’m upset, I feel all alone in the world”). The refined model had an acceptable fit to the data (χ^2^(df = 51) = 255.56, *p* < 0.001, RMSEA = 0.08, CFI = 0.94, TLI = 0.93, SRMR = 0.04). The refined model is represented in the left part of Figure 1.

### 3.2. Psychometric Properties of the EDS

The EDS-s had satisfactory internal consistency (ordinal alpha = 0.94, MS = 0.93, and LCRC = 0.93). The average score was 37.88 (SD = 15.65) with significant differences between men and women (t_4.926_ = −4.97, *p* = 0.027, Cohen’s d = 0.33). Men had lower mean scores (=34.36, SD = 14.19) than women (=39.49, SD = 16.02). The ANOVA revealed significant differences among the age groups (F = 9.811, df = 21,084, *p* < 0.001). Both the young adults (age 18–34) and the adults (35–49) reported higher mean scores (M = 39.50, SD = 15.65, *p* < 0.001, Cohen’s d = 0.33; and M = 38.26, SD = 15.99, *p* = 0.029, Cohen’s d = 0.24, respectively) compared to late adults (M = 34.53, SD = 14.98). No floor or ceiling effects were detected.

The adequacy of the refined factor model was tested using SEM regression analysis with the BES and BSI as the dependent variables, while controlling for age and sex. The regression model reported an adequate fit to the data: χ^2^ = 234.87, df = 99, *p* < 0.001, RMSEA = 0.06, CFI = 0.97, TLI = 0.97, SRMR = 0.04. The EDS-s explained 60% of the variance of the GSI total score (*p* < 0.001; 99% CI: 0.46–0.71) and 35% of the variance of the BES (*p* < 0.001; 99% CI: 0.19–0.48) (Figure 1).

## 4. Discussion

Although the current study confirmed the unidimensionality of the EDS-s, we had to include correlations between errors for three pairs of items (#1 with #10; #11 with #12; #5 with #7), a decision also supported theoretically [41]. Thus, the variance in common between these pairs of items was not only related to the common factor, expressing emotion dysregulation, but also to artifacts due to the wording of the items. However, some authors do not agree with the practice of correlating items’ errors because it can lead to issues with interpretation of the model [42].

As reported by other studies [18,20,23], the EDS-s has a high internal consistency (ordinal alpha = 0.94). Sex differences were observed, with females reporting higher EDS-s scores, indicating greater difficulties in modulating the expression of negative emotions compared to males [43,44]. Moreover, the fact that more women took part in the current study is probably due to the fact that women are more inclined to take part in surveys than men. Lastly, the higher percentage of women in our study could have affected sex differences in emotion dysregulation. Significant differences were found for age as well. Young adults reported the highest scores when compared to adults and late adults. This result is also in line with previous studies reporting a greater ED associated with younger age, probably due to the fact that, as people get older, they learn to cope with stressors and avoid emotionally-triggering situations [45,46].

Our results demonstrated the convergent validity of the EDS-s with binge eating symptomatology and general psychopathology, which is consistent with previous studies [47,48]. These findings are in line with the hypothesis that experiencing dysregulated negative emotions may trigger binge eating behavior, which could be used as a coping strategy for heightened emotional dysregulation [47,49]. Moreover, studies on children and adolescents demonstrated that the presence at a younger age of emotional dysregulation is a significant risk factor for later psychopathology [48], but not the other way around [50]. These results suggest that alterations in neuroendocrine functioning and/or neural pathways may be linked to individual differences in the central nervous system (CNS), which in turn may increase the risk of psychopathology [50,51]. However, the mechanisms by which emotional dysregulation and general psychopathology are related are still not clear [50].

The limitations of this study that affect the generalizability of the results need to be taken into account. First, due to the correlational nature of the study, no causal interpretation can be inferred from the mediation analysis. Second, the high number of women in our sample does not correspond to the actual women/men ratio in the general population. This is probably due to the fact that women are more inclined to take part in surveys than men. Moreover, a higher percentage of women could have had an impact on sex differences in emotion dysregulation. Third, we did not administer an alternative measure of ED.

## 5. Conclusions

The EDS-s is a unidimensional and reliable tool for assessing emotion dysregulation. Future studies are needed to investigate the stability of the dimensionality of the EDS-s over time. Moreover, studies in clinical samples are also needed to investigate the stability of the factor structure of the EDS-s across different psychiatric diagnoses and conditions. The availability of a short instrument could help clinicians save time in the assessment procedure, and it could also be helpful to researchers as a brief emotional dysregulation questionnaire in large epidemiological cohort studies and in clinical samples with eating disorder symptoms. Lastly, for this reason, it is important for both clinicians and researchers to have free access to such diagnostic tools.

## Figures and Tables

**Table 1:** Descriptive statistics of the sample (*n* = 1087).

Variables	Count/M	%/(SD)
Age M(SD)	36.02	(15.56)
Gender *n*/%		
Men	319	29.3%
Women	768	70.7%
School attainment ≥ 13 years *n*/%	607	55.8%
Job status *n*/%		
Employed	513	47.2%
Unemployed	207	19.0%
Student	363	33.4%
Marital status *n*/%		
Married or in a stable relationship	386	35.5%
Single	693	63.8%
EDS-s M(SD)	37.98	(15.67)

M = Mean; SD = Standard Deviation; % = Percentage; EDS-s = Emotion Dysregulation Scale-short.

**Table 2:** Descriptive statistics of the subsample considered for the regression analysis (*n* = 295).

Variables	Count/M	%/(SD)
Age M (SD)	36.17	(15.17)
Gender *n*/%		
Men	114	38.6%
Women	181	61.4%
School attainment ≥ 13 years *n*/%	125	42.4%
Job status *n*/%		
Employed	162	54.9%
Unemployed	34	11.5%
Student	99	33.6%
Marital status *n*/%		
Married or in a stable relationship	112	38.0%
Single	183	62.0%
EDS-s M(SD)	35.68	(14.94)
BES M(SD)	8.29	(6.98)
GSI M(SD)	34.21	(26.38)

M = Mean; SD = Standard Deviation; % = Percentage; EDS-s = Emotion Dysregulation Scale-short; BES = Binge Eating Scale; GSI = Global Severity Index of the Brief Symptom Inventory.

## Data Availability

The data presented in this study are available from the corresponding author on reasonable request.

## Appendix A. Italian Version of the EDS-s

**Table A1:** Istruzioni: Per favore valuti in che misura ciascuna delle affermazioni che seguono la descrive, dove.

	Per Nulla Vero			In Parte Vero			Assolutamente Vero
1. Mi è spesso difficile calmarmi quando sono agitato	1	2	3	4	5	6	7
2. Quando sono agitato, ho difficoltà a capire quello che provo, mi sento solo male	1	2	3	4	5	6	7
3. Quando mi sento male, ho difficoltà a ricordare qualcosa di positivo, tutto sembra negativo	1	2	3	4	5	6	7
4. Le emozioni mi travolgono	1	2	3	4	5	6	7
5. Quando sono agitato, mi sento solo al mondo	1	2	3	4	5	6	7
6. Quando sono agitato, ho difficoltà a risolvere i problemi	1	2	3	4	5	6	7
7. Quando sono agitato, ho difficoltà a ricordare che ci sono persone che si preoccupano per me	1	2	3	4	5	6	7
8. Quando sono agitato, tutto appare come catastrofico o difficile	1	2	3	4	5	6	7
9. Quando sono agitato, ho difficoltà a vedere o ricordare qualcosa di buono su di me	1	2	3	4	5	6	7
10. Quando sono agitato, ho difficoltà a calmarmi	1	2	3	4	5	6	7
11. Quando provo emozioni forti, ho difficoltà a pensare in modo chiaro	1	2	3	4	5	6	7
12. Quando provo forti emozioni, prendo spesso delle cattive decisioni	1	2	3	4	5	6	7

1 = per nulla vero, 4 = in parte vero, e 7 = assolutamente vero.
